# Stereotactic Ablative Radiotherapy Combined with Immune Checkpoint Inhibitors Reboots the Immune Response Assisted by Immunotherapy in Metastatic Lung Cancer: A Systematic Review

**DOI:** 10.3390/ijms20092173

**Published:** 2019-05-02

**Authors:** Rodolfo Chicas-Sett, Ignacio Morales-Orue, Juan Castilla-Martinez, Juan Zafra-Martin, Andrea Kannemann, Jesus Blanco, Marta Lloret, Pedro C Lara

**Affiliations:** 1 Department of Radiation Oncology, “Dr. Negrín” University Hospital of Gran Canaria, Barranco de la Ballena s/n, 35010 Las Palmas de Gran Canaria, Spain; ignaciomorue@gmail.com (I.M.-O.); juanfe4206@hotmail.com (J.C.-M.); jzafra08@gmail.com (J.Z.-M.); andreakannemann@hotmail.com (A.K.); blancosuar@hotmail.com (J.B.); mllosae@hotmail.com (M.L.); 2 Clinical Oncology, Medical School, Las Palmas University of Gran Canaria, 35001 Las Palmas de Gran Canaria, Spain; 3 Department of Oncology, San Roque University Hospital, Calle Dolores de la Rocha 5, 35001 Las Palmas de Gran Canaria, Spain; pedrocarlos.lara@ulpgc.es; 4 Faculty of Health Sciences, Fernando Pessoa Canarias University, Calle la Juventud S/N, 35450 Las Palmas, Spain

**Keywords:** abscopal effect, radiotherapy, immunotherapy, CTLA-4, Anti-PD-1/PD-L1, SABR, ICI, SBRT

## Abstract

*Background:* Immune checkpoint inhibitors (ICI) have represented a revolution in the treatment of non-small-cell lung cancer (NSCLC). To improve these results, combined approaches are being tested. The addition of stereotactic ablative radiotherapy (SABR) to ICI seems promising. A systematic review was performed in order to assess the safety and efficacy of SABR-ICI combination. *Material and Methods:* MEDLINE databases from 2009 to March 3, 2019 were reviewed to obtain English language studies reporting clinical outcomes of the combination of ICI-SABR in NSCLC. 18 out of the 429 initial results fulfilled the inclusion criteria and were selected for review. *Results:* Eighteen articles, including six prospective studies, describing 1736 patients treated with an ICI-SABR combination fulfilled the selection criteria. The reported mean rates for local control and distant/abscopal response rates were 71% and 41%, respectively. Eleven studies reported progression-free survival and overall survival, with a mean of 4.6 and 12.4 months, respectively. Toxicity rates were consistent with the ones attributable to ICI treatment alone. *Conclusions:* The ICI-SABR combination has a good safety profile and achieves high rates of local control and greater chances of obtaining abscopal responses than SABR alone, with a relevant impact on PFS. More studies are needed to improve patient selection for an optimal benefit from this approach.

## 1. Introduction

### 1.1. Lung Cancer and SABR

Lung cancer is the second most frequently diagnosed tumor worldwide and is the leading cause of cancer-related deaths [1]. No significant changes in 5-year survival have been observed in the last three decades [2,3].

Radiation therapy (RT) has a role in all stages of non-small-cell lung cancer (NSCLC), including definitive therapy in the oligometastatic setting using SABR [4]. SABR is defined as a RT schedule that uses high (i.e., ablative) doses per fraction in one to five fractions with highly conformal techniques [5]. SABR has been established as a standard of care for patients with early-stage NSCLC who are medically inoperable or who refuse surgery [6,7,8,9,10]. These favorable results have been translated to the oligometastatic setting, where SABR has been adopted as standard treatment for patients not eligible for surgery by improving progression free survival (PFS) and overall survival (OS) [11,12,13,14,15,16,17,18,19,20,21,22,23,24]. Gomez et al. [24] reported a high median PFS of 11.9 months after local consolidative radiotherapy (including SABR) in patients with oligometastatic NSCLC involving ≤3 sites that had not progressed after first line chemotherapy.

### 1.2. The Use of Immune Checkpoint Inhibitors (ICI) in Lung Cancer

NSCLC has traditionally been considered a poorly immunogenic tumor [25,26]. Recent advances in the field of immunotherapy, targeting down-regulators of the immune system with different therapeutic agents generically known as immune checkpoint inhibitors (ICI), have shown promising results and are currently establishing a new standard of care in advanced NSCLC [27,28]. The two most therapeutically relevant checkpoints are cytotoxic T-lymphocyte-associated antigen 4 (CTLA-4) and programmed cell death protein-1 (PD-1). Monoclonal antibodies targeting these checkpoints include nivolumab, pembrolizumab (anti-PD-1), atezolizumab, durvalumab (anti-PD-L1) and ipilimumab (anti-CTLA-4). Their goal is to induce immune cell proliferation and activation against cancer cells by “taking off the brakes” of the immune system [29].

The effectiveness of nivolumab in NSCLC was confirmed by two large phase III trials, CheckMate 017 [27] and CheckMate 057 [28], leading to its approval as second-line treatment in advanced NSCLC in October 2015. However, these favorable results for nivolumab monotherapy as second-line treatment were not translated to first-line setting. The CheckMate-026 trial compared nivolumab to standard-of-care chemotherapy in first-line treatment of advanced or recurrent NSCLC with PD-L1 positivity ≥5% and did not demonstrate a significant survival benefit for nivolumab [30].

The efficacy of pembrolizumab was confirmed by the results of the phase II/III trial KEYNOTE-010, which enrolled patients with previously treated advanced NSCLC and PD-L1 expression on at least 1% of tumor cells and compared OS and PFS for three treatment arms: pembrolizumab (two arms, 2 or 10 mg/kg) or docetaxel (one arm, 75 mg/m^2^). In the total population, both pembrolizumab arms had significantly improved OS compared with the docetaxel arm. Among the subgroup of patients with PD-L1 expression PS ≥50%, either dose of pembrolizumab significantly improved OS in comparison to docetaxel. Despite these differences in OS, no difference was found in PFS between the three study arms. Pembrolizumab was better tolerated than docetaxel, with grade ≥3 adverse events in 13%–16% vs. 35% of patients [31].

Atezolizumab was assessed in POPLAR, a phase II trial, comparing atezolizumab with docetaxel in patients with previously treated advanced NSCLC, stratifying patients according to PD-L1 status, histologic type and previous lines of therapy. In the intention-to-treat population, atezolizumab significantly improved median OS compared with docetaxel. PD-L1 expression on tumor cells or tumor-infiltrating immune cells was associated with an OS benefit [32].

Ipilimumab, a CTLA-4 antagonist used in monotherapy in advanced NSCLC, had previously shown limited activity [33]. However, results are promising when combined with cytotoxic chemotherapy. A phase II trial comparing carboplatin/paclitaxel with or without ipilimumab as first-line treatment for patients with advanced NSCLC showed that the addition of ipilimumab significantly improved PFS [34].

Even though these drugs have shown remarkable action against several types of tumors, only 20% of patients obtain a clinical benefit [35]. Consequently, it is extremely important to study additional treatment alternatives intended to improve response rates.

### 1.3. Rationale of Radiotherapy and ICI Combination

RT has shown an immunomodulating effect, instigating a localized stimulation of the immune system by increasing antigen presentation (auto-vaccination) through immunogenic cell death (ICD) [36], reducing myeloid-derived suppressor cells (MDSCs) [37], modifying macrophage polarization [37], increasing PD-1 tumor expression and stimulating vascular modification [38], all of which have the potential to modify the tumor microenvironment [39,40]. This facilitates a reinvigoration of the immune response, increasing the chances of achieving an abscopal effect; a term coined by Mole in 1953 describing an event in which focalized RT unleashes systemic anti-tumoral action that can result in distant responses [41].

This elusive effect begets new challenges with the widespread use of ICI, encouraging investigators to explore novel therapeutic approaches such as, a) adjusting radiation doses to take full advantage of the potential for immune stimulation, b) defining the most advantageous sequencing for radiation, c) determining the ideal drugs to use alongside radiation, and d) counteracting the immunosuppressive components concerned [42].

Available data suggests that the combination of RT and ICI may be a feasible and safe strategy, with reports of up to 26.5% increased abscopal responses and an 8-month increase in overall survival in melanoma patients treated with this association [42].

## 2. Materials and Methods

### 2.1. Search Strategy

We reviewed MEDLINE (via PubMed) databases from 2009 to March 3, 2019 in order to obtain English language studies reporting clinical outcomes of the combination of RT with ICI in NSCLC. Several terms were used, including “radiotherapy”, “immunotherapy”, “lung cancer”, “SABR”, “SRS”, “SBRT”, “immune checkpoint inhibitors”, “anti-CTLA-4”, “anti-PD-1”, “anti-PD-L1”, “abscopal effect”. Non-original articles were excluded.

### 2.2. Selection of Studies and Data Compilation

All articles were assessed based on title and abstract. The included studies relevant for this review met the following criteria:

a) Combined RT and sequential or concurrent ICI treatment.

b) External beam RT.

c) Study type included prospective or retrospective.

d) Studies published in English.

### 2.3. Statistical Considerations

Outcomes were analyzed in terms of local control, distant/abscopal response rates, PFS, OS and Toxicity > Grade 3. To analyze distant/abscopal response rates, PFS, OS and toxicity, we pooled the results of each article and calculated a weighted mean (WM) between them. The criteria used were randomization (randomized studies were more highly weighted than non-randomized ones and than other categories), study design (prospective studies were more highly weighted than retrospective studies, which were classified in weight as *n* >30 or *n* <30, respectively) and sample size. The weighted mean was defined by the following formula [43]:

VM = ∑*^n^*_i=1_ w_i_Z*i*/∑*^n^*_i=1_ w_i_ (1)

where *n* is the sample size and w_i_ is the weighting factor of the i^th^ observation (i = 1..., *n*).

## 3. Results and Discussion

**Figure 1:**
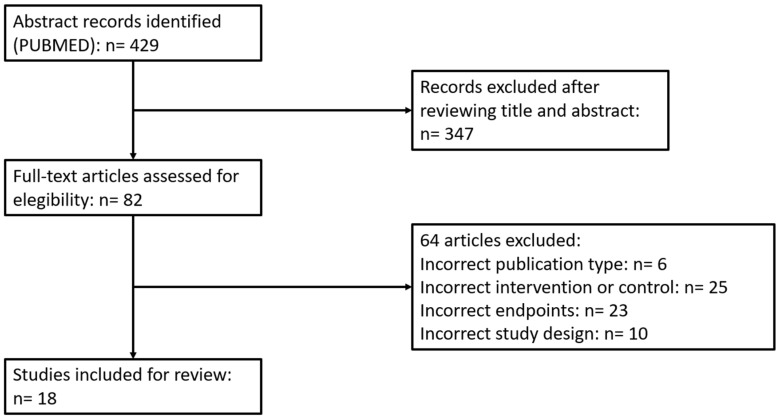
Flow chart of systematic literature search process according to PRISMA statement.

Our search generated a total of 429 results. Through a process of screening, 18 publications were selected for the review. Out of the 411 studies excluded for this review, 347 were excluded due to not fulfilling the specific inclusion criteria, 8 because of inadequate publication type, 24 due to incorrect intervention or control, 22 did not include the correct endpoints, and 9 were excluded because of an incorrect study design. Therefore, 18 fulfilled the inclusion criteria and were included in our review. The flowchart detailing the systematic literature search process is shown in Figure 1.

These 18 studies included a total of 1736 patients, and in 6 of them the study design was prospective (Table 1).

### 3.1. Is It Possible to Obtain Target Control with the Combination of RT and ICI?

Data on local control (LC) was available for only 3 studies (399 patients), 1 being phase I and 2 being retrospective. Overall, the weighted mean LC rate was 70.7%, ranging from 64% to 90% [46,51,59].

It must be noted that the intent of SABR-ICI treatment is to change the natural history of the disease by targeting metastatic lesions and, as a secondary objective, to improve locorregional control using lower SABR doses.

At present, SABR is the standard treatment for non-operable NSCLC, with LC rates ≥90% when administering doses ≥100 Gy BED_10_ [62]. In this context, high SABR doses combined with ICI have been evaluated by Tang et al. [46] in a phase I trial. 14 patients were treated with SABR doses of 50 Gy in 4 fractions, reporting a similar LC rate (90%). Conversely, a similar LC rate can be observed with the use of lower SABR doses combined with ICI [51,59], as summarized in Table 1. This situation can be explained by the radio-sensitizing effect of ICI and the immunogenic effects of SABR, such as induction of ICD, release of tumor-associated antigens, alteration of target cell immunophenotype and modulation of the TME [63].

Additionally, an effective LC rate could play a fundamental role in metastatic patients who develop isolated sited progression while on ICI, enabling a sustained and more durable systemic response [64]. For example, Pike et al. [65] reported that the use of radiotherapy in oligoprogression allowed for the continuation of anti-PD-1 treatment, while maintaining a systemic response in 23 of 59 patients.

### 3.2. Using RT-ICI Combination to Improve Systemic Immunotherapy Effects: Abscopal Response in NCSLC

Eight out of the 18 studies included in this review [44,45,46,47,48,49,50,60] assessed and reported the systemic response observed. It is important to note that only two studies were specifically designed to evaluate the abscopal response as a primary endpoint in NSCLC [44,48], while the rest of the studies reported abscopal responses as the systemic response rate according to RECIST criteria.

**Figure 2:**
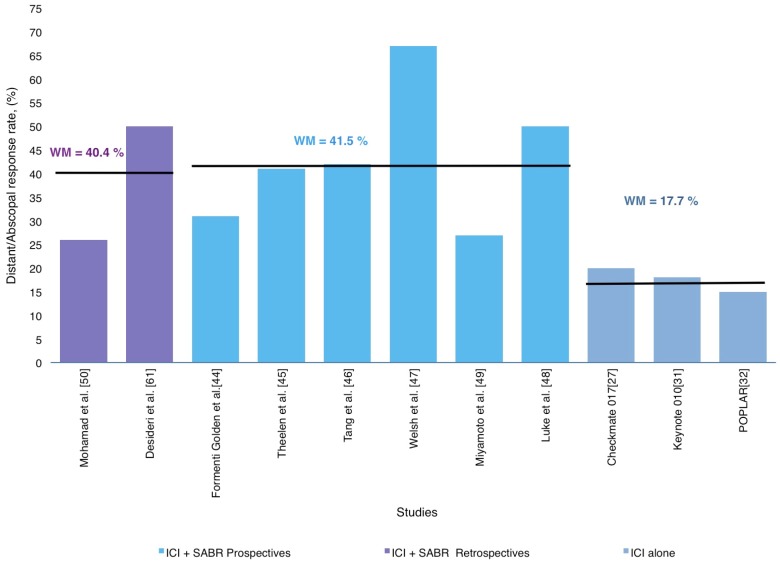
Distribution of weighted mean distant/abscopal response rates according in prospective and retrospective studies in SABR-ICI combination and ICI alone trials.

Overall, the distant/abscopal response was reported in a cohort of 412 patients with a weighted mean of 41.3%, ranging from 26.0% to 67.0%, as described in Table 1 and Figure 2.

Similar distant/abscopal responses were found among retrospective and prospective studies (34.0% and 42.8%, respectively), but better than studies with ICI alone (15%–44%) [27,28,30,31,32]. Figure 2 represents an approximate distribution of the distant/abscopal response rate reported in all studies included in this review. For instance, the phase II trial by Formenti et al. [44] included patients with chemo-refractory metastatic NSCLC who received ipilimumab concurrently with SABR (30 Gy in 5 fractions) to a single metastatic lesion. Abscopal response was 31% including CR, PR and SD, whereas the median OS for survivors was 43 months (range: 38–47 months). Luke et al. [48] also reported an abscopal response rate of 26%. As mentioned above, most studies included in this section showed promising outcomes regarding systemic disease control, but their heterogeneity in relation to primary histology and radiotherapy fractionation represents a weak point in the interpretation of their results. To help answer the question of whether SABR and ICI combination increases systemic response in metastatic NSCLC patients, Theelen et al. [45] performed a randomized phase II trial that included 74 patients with advanced NSCLC (>2^nd^ Line) which were randomized (1:1) to receive pembrolizumab (200 mg q3w) alone or pembrolizumab preceded by SABR (8 Gy × 3 fractions within 7 days prior to the first cycle) on a single metastasis. The overall response rate was 19% in the control arm (pembrolizumab alone) and 41% in the SABR-pembrolizumab arm. Thus, this trial concluded that SABR-pembrolizumab treatment resulted in a doubling of overall response rate while maintaining a good safety profile.

### 3.3. The Abscopal Response Potentially Delays Disease Progression

**Figure 3:**
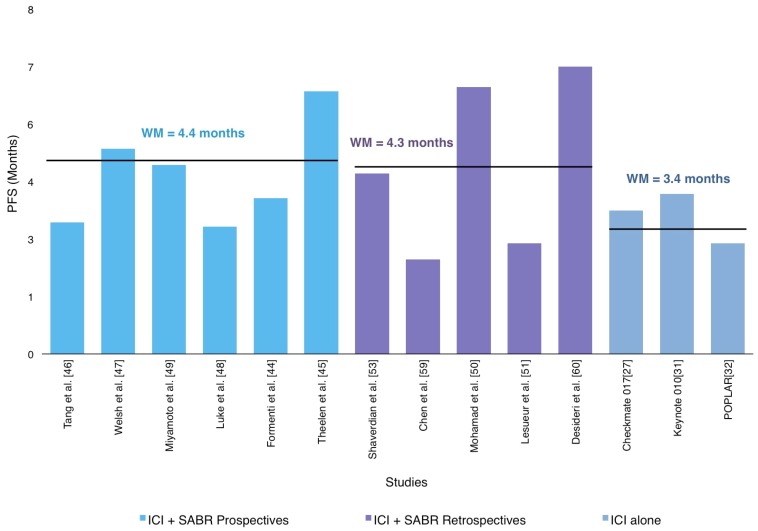
Distribution of weighted mean progression-free survival (PFS) according in prospective and retrospective studies in SABR-ICI combination.

PFS data was available in 818 patients included in 11 reviewed trials [44,45,46,47,48,49,50,51,52,58,59]. The weighted PFS mean was 4.6 months (2.3–7 months), as shown in Figure 3.

Although the PFS reported in the studies included in this review has a wide variation regardless of the nature of its design (retrospective or prospective), our results were consistent with the major studies that had previously evaluated the use of ICI in monotherapy [27,28,30,31,32] as described in Figure 3. On the other hand, prospective trials, have reported higher PFS with the combination of RT and ICI. One of them is a randomized phase II trial that reported an improvement in PFS: 6.4 months in the SABR-Pembro arm compared with 1.8 months in the Pembro alone arm [43]. In addition, a secondary analysis of the phase I KEYNOTE-001 trial, with a median follow-up at 32.5 months, showed that 43% (*n* = 42) of patients who received extracranial irradiation before pembrolizumab reported significantly longer PFS than those without prior RT (4.4 vs 2.1 months; *p* = 0.01) [52], suggesting the potential of radiotherapy to turn traditional non-responders into responders.

Thus, all of the above suggests that select patients with metastatic NSCLC may be appropriate candidates for this new promising treatment approach.

### 3.4. Could Radiotherapy and ICI Combination Improve Overall Survival in Metastatic NSCLC Patients?

**Figure 4:**
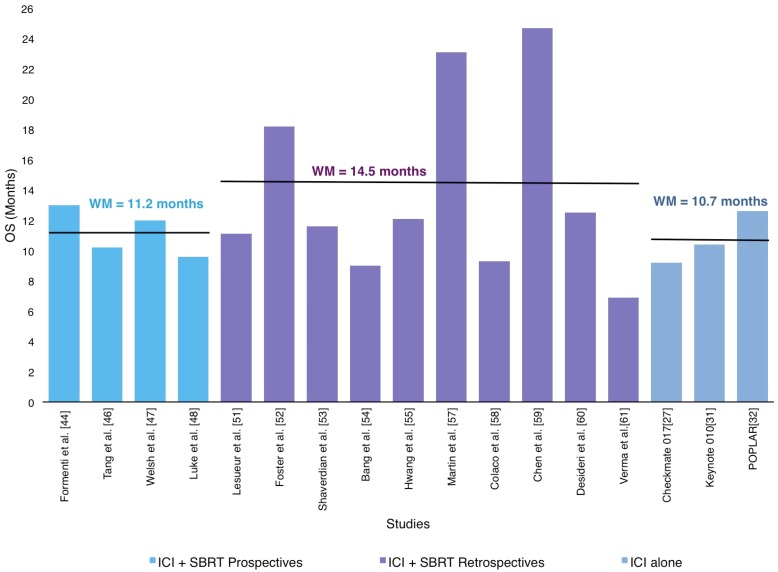
Distribution of weighted mean overall survival (OS) according in prospective and retrospective studies in SABR-ICI combination and ICI alone studies.

11 out of the 18 articles included in this review [44,46,47,48,51,52,53,55,59,60] reported the median OS of the subjects accrued in their respective studies. The weighted OS mean for the 1310 patients was 12.4 months (9.0–24.7 months), as shown in Figure 4.

A similar benefit with RT-ICI combination was observed in comparison with the reported OS in the phase II and III trials of anti-CTLA-4 and anti-PD-1/PD-L1 without RT [27,28,30,31,32]. The most relevant studies reporting OS are described below and summarized in Table 1.

Given that the majority of these studies include unselected patients who had been heavily treated beforehand, interpretations on OS findings should be taken with caution. For this reason, an analysis of the main factors related to OS is crucial. Therefore, in this review we have taken into consideration variables such as timing of RT administration, dose and fractionation, irradiation site and tumor burden [51,52,53,55,58,59,60].

Regarding the timing of RT treatment, we observed contradictory data. For example, Sharvedian et al. [53], in his retrospective analysis of the results of the phase 1 KEYNOTE-001 trial, found that median OS was higher in patients with prior RT (10.7 months vs 5.3). Hwang et al. [55] performed a retrospective cohort study of 164 patients with metastatic lung cancer treated with PD-1/PD-L1 inhibitors, finding a median OS of 12.1 months and reduced all-cause mortality in patients who received RT before ICI initiation. Chen et al. [59] retrospectively evaluated 260 patients (157 with NSCLC) treated with ICI (ipilimumab, nivolumab or pembrolizumab) and concurrent or sequential SRS/SABR (*n* = 79), finding that SRS-SABR with concurrent ICI was associated with improved OS compared with both SRS-SABR alone (24.7 vs 12. 9 months, *p* = 0.002; hazard ratio, 2.69) and non-concurrent SRS-SABR and ICI (*p* = 0.006; hazard ratio, 2.40). On the other hand, Lessuer et al. [50], in a retrospective study of a multicentric analysis on the safety and efficacy of concurrent or sequential ICI and hypofractioned-RT, found that the OS did not seem to be associated with the timing of RT administration.

In regard to RT doses, Foster et al. [52] retrospectively analyzed a national cancer database that included stage IV NSCLC patients receiving chemotherapy or immunotherapy. RT modality was classified as SABR to intra- and/or extracranial sites. Two findings of this study are particularly interesting: 1) median OS was 18.2 months for SRS + ICI compared to 14.3 months for SRS + chemotherapy, and 2) SRS with biologically effective dose (BED) >60 Gy was independently associated with improved OS.

RT location and local control has also been associated with OS in patients with RT-ICI treatments. Colaco et al. [58] included 180 patients (71 with NSCLC) who underwent GammaKnife SRS with sequential chemotherapy, targeted therapy or ICI. Median OS was significantly longer in patients who developed local response (23.7 vs 9.9 months, respectively).

Lastly, Desideri et al. [60] published a retrospective analysis that included 20 patients with metastatic NSCLC (*n* = 17) and RCC who received concomitant nivolumab and RT. Oligoprogressive patients treated with ablative intent, compared to patients undergoing RT with palliative-only intent, had statistically longer OS (17.9 vs 10.3 months, HR 0.41 CI 0.16–1.02, *p* = 0.04).

In short, our review shows that a clinical benefit in OS when using RT-ICI combination remains unclear. This could be attributed to the heterogeneity of these studies, as well as the fact that patient follow-up is still limited.

### 3.5. Radiotherapy and ICI: A Safe Combination

Immunotherapy has a different and often better-tolerated profile of adverse effects (AEs) than chemotherapy. Toxicity from ICI usually includes fatigue, rash, myalgias and pneumonitis. Even though these side effects are generally mild, they can also be potentially severe. The evidence available regarding these AEs comes from three main trials: Checkmate 017, Keynote 010 and POPLAR, which found grade ≥3 irAEs of 7%, 13%–16% and 11%, respectively when administered in monotherapy [27,28,30,31,32], as described in Table 2.

Evidence on the combination of immunotherapy with radiotherapy is still limited, but the data available suggests that toxicity derived from the combined treatment does not increase in comparison to immunotherapy alone [27,28,30,31,32] Grade ≥3 toxicity from the RT/ICI combination ranges between 10%–17% for anti PD-1/PD-L1 and 29%–38% for anti CTLA-4, according to the 6 prospective studies that we found (Table 1). These numbers are consistent with the AEs than can be expected from ICI treatment alone [27,28,30,31,32]. The only exception is the phase III study by Govindan et al. [66], which describes a grade ≥3 toxicity of 53%. However, this trial included chemotherapy in addition to ipilimumab, which might explain these increased side effects.

**Figure 5:**
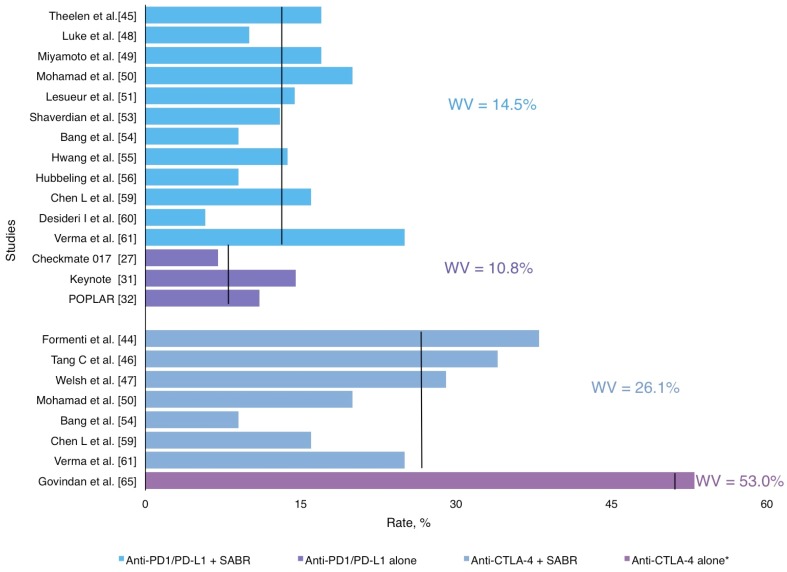
Grade ≥3 Adverse Events in SABR and ICI combination treatment.

It must be noted, however, that few studies randomize patients between ICI monotherapy and ICI plus radiotherapy. Moreover, in the studies where RT is sequential to immunotherapy, basal toxicity from ICI is rarely mentioned [45,46,47,48,49]. Registered toxicity with the combined treatment can be seen in Figure 5.

Still, out of the 6 prospective studies, the phase II trial by Theleen et al. [45] did include a control arm of ICI alone. This study compared the use of pembrolizumab with or without radiotherapy in NSCLC. Their results showed grade ≥3 toxicity of 22% in the pembrolizumab arm versus 17% in the pembrolizumab plus radiotherapy arm. Furthermore, the PACIFIC III trial showed comparable grade ≥3 toxicity between the ICI and control groups, even though this study did not include metastatic patients, but did include patients with locally advanced stage 3 NSCLC [67].

Therefore, the combination of radiotherapy and immunotherapy seems to be safe. However, some studies do report increased grade ≥3 toxicity. In particular, three trials testing the combination of ipilimumab with radiotherapy presented 34% (Tang et al.), 29% (Welsh et al.) and 38% (Formenti et al.) grade ≥3 toxicities [44,46,47]. It must be noted that the first study did not specify which toxicities were attributable to the combined treatment, while the second and third trial showed that only 2% and 10% respectively were caused by the combination of ICI and RT. Furthermore, these studies include patients treated with chemotherapy in addition to ICI.

As stated before, evidence of AEs from prospective trials is still limited. However, there are a growing number of retrospective studies that may provide useful data. Grade ≥3 toxicities in these studies range from 5.8% to 25%, which is consistent with historical controls and further supports the idea that the combination of ICI and radiotherapy is safe (Figure 5). Again, a number of patients also received treatment with chemotherapy, which can explain the increased rate of AEs in some of these studies.

Although the available evidence suggests that RT does not increase the side effect profile of immunotherapy, there are still many questions regarding if different treatment-related variables can have an influence on the severity of these toxicities.

One increasingly important subject involves treatment timing and sequencing. In the prospective clinical trials that we reviewed, radiotherapy was administered either concurrently or within seven days of the first dose of ICI, with the exception of Miyamoto et al. [49], where treatment with nivolumab started within two weeks of the first fraction of SBRT. Moreover, patients in the PACIFIC trial were randomized 1–42 days after chemo-radiotherapy. In the trial by Tang et al. [46], patients receiving a sequential treatment presented grade ≥3 toxicity of 25%–50% in comparison to 29%–33% in the concurrent treatment group. However, in this study only 8 of the 35 patients had lung cancer and the toxicity of this specific group was not described. The rest of the prospective studies do not report differences in toxicity.

Regarding the retrospective studies, most of them do not analyze specific differences in toxicity according to timing. In the ones that delivered both concurrent and sequential treatments, all but one study found no significant differences in toxicity. The study by Bang et al. [54] did describe a trend towards higher overall toxicity when radiation was administered within 14 days of immunotherapy (39% vs 23%, *p* = 0.06). However, no significant differences in grade ≥3 AEs were observed.

Further prospective studies are needed to determine if the sequence or interval between treatments might have an impact on toxicity.

A fundamental matter regarding AEs has to do with follow-up in the long term. Immune-induced side effects can appear months after treatment, while toxicity from RT may emerge even years later. At the moment, given that the prospective trials available are very recent, follow-up is still very short. The exception is the study by Formenti et al. [44], which has a median follow-up of 43 months. As for retrospective studies, follow-up ranges between 6–32 months. For this reason, further studies are needed in order to verify that toxicity profiles do not increase over time.

In regard to other variables such as RT fractionation or treatment site, there is not enough data available to draw conclusions on how they might affect toxicity profiles. Specific differences in toxicity between radiotherapy schedules were not analyzed in any of the studies. Authors like Bang et al. [54] describe a relation between overall toxicity and BED. However, grade ≥3 AEs do not seem to increase with high BED.

Furthermore, most of the studies do not specify toxicity rates by treatment site. Bang et al. [54] found no significant link between site-specific immune toxicity and radiation administered in the corresponding anatomical area. In any case, future prospective trials should shed some light on these matters.

As a whole, the combination of ICI and radiotherapy seems to be comparable with ICI monotherapy in terms of toxicity. However, it must be noted that the available data is still preliminary and most of this data does not come from prospective trials. Moreover, evidence on how treatment variables might affect toxicity profiles is lacking, although the results from the present studies generally have not found significant differences. Future prospective studies are needed to confirm these findings.

### 3.6. Futures Perspectives

Immunotherapy has represented a paradigm shift in the treatment of NSCLC. Due to its positive results in recent studies, there is a trend towards treating a growing number of patients with ICI. For this reason, finding out which patients will benefit the most from these treatments is becoming essential. The addition of other therapies to ICI seems to be a good way of getting closer to an optimal treatment. As it has been stated in this review, RT in particular is a very promising approach, and novel ways of delivering these treatments are constantly in the works. For instance, a recent review described how nanoparticles have the potential to enhance abscopal responses in an RT-ICI combination [68]. All these new approaches, along with future prospective trials, will help determine the best way of administering what is shaping up to be a very promising therapy.

## 4. Conclusions

Even though several studies showed an increase in median OS and PFS, a pooled analysis of these studies does not indicate a general increase in median OS and PFS, observing similar results to the ICI alone baseline studies described in this article. On the other hand, a consistent outcome among all studies is an increased distant/abscopal response rate in patients treated with SABR and ICI, suggesting the potential of radiotherapy to turn traditional non-responders into responders. Why these clear clinical outcomes advantages do not translate into OS and PFS would be explained, for the heavily pretreatment that precludes further systemic treatment after progression to ICI+RT. With the current scientific evidence provided, this treatment combination looks like a promising therapeutic approach, and further prospective studies could shed some light on the ideal treatment schedule involving ICI association and optimal RT timing, dosage and fractionation.

## Figures and Tables

**Table 1:** Prospective and retrospectives trials reporting clinical results of radiotherapy and ICI combination in metastatic NSCLC.

Author	Study Type	N	Cancer Histology	RT Target	RT Dose Gy/Fraction	Treatment Sequencing	IT Agent	IT Dose	Local Control Rate (CR+PR+S) %	Median OS (months)	PFS (months)	Distant/Abscopal Response Rate (CR+PR+S)%	Toxicity ≥ Grade 3 (%)
Formenti et al. 2018 [44]	Phase I-II	39	NSCLC	NR	28.5–30/ 3–5	Concurrent	Ipi	3 mg/Kg/ 3w	NR	13	3.8	31% (Abscopal)	38
Theelen et al. 2018 [45]	Phase II	74	NSCLC	NR	24/3	Sequential	Pembro	200 mg q3w	NR	NR	6.4	41%	17
Tang et al. 2017 [46]	Phase I	35 Lung: 14	Various	Lung, liver	50/4	Concurrent Sequential	Ipi	3 mg/Kg/ 3w	90%	10.2	3.2	42%	34
Welsh et al. 2017 [47]	Phase II	100	Various	Lung, liver	50/4	Concurrent Sequential	Ipi	3 mg/Kg/ 3w	NR	12	5.0	67%	29%
Luke et al. 2018 [48]	Phase I	79 Lung: 7	Various	Lung, liver, bone, abdomen, pelvis	30–50/ 3–5	Sequential	Pembro	200 mg q3w	NR	9.6	3.1	26.9% (Abscopal) Systemic: 13%	10
Miyamoto et al. 2018 [49]	Prospective	6	NSCLC	Lung	25–48/ 3–4	Sequential	Nivo	3 mg/kg q2w	NR	NR	4.6	50%	17
Mohamad et al. 2018 [50]	Retrospective	59 Lung; 5	Various	Extracranial	>5 Gy perfx/ 1–5	Concurrent Sequential	Nivo, Pembro Atezo, Ipi	NR	NR	Not reached	6.5	26%	20
Lesueur et al. 2018 [51]	Retrospective	104	NSCLC	Bone, brain, lung, others	RT3D: 20–30/ 5–10 SABR: 20–36/ 1–6	Concurrent (*n* = 45) Sequential (*n* = 59)	Nivo	NR	2 y-LC: 64.4%	11.1	2.7	NR	14.4
Foster et al. 2019 [52]	Retrospective	228	NSCLC	Intracranial	18–50/ 1–5	NR	NR	NR	NR	18.2	NR	NR	NR
Shaverdian et al. 2017 [53]	Retrospective	42	NSCLC	Thoracic	NR	Sequential	Pembro	2 mg/kg or 10 mg/kg q3w; 10 mg/kg q2w	NR	11.6	4.4	NR	13
Bang et al. 2017 [54]	Retrospective	133 Lung: 71	NSCLC	Lung, bowel, brain, neck	8–37.5/ 1–15	Concurrent Sequential	Anti-CTLA-4 Anti-PD-1	NR	NR	NR	NR	NR	9
Hwang et al. 2017 [55]	Retrospective	164	NSCLC SCLC	Lung	8–60/ 1–30	Concurrent Sequential	Anti-PD-1 Anti-PD-L1	NR	NR	12.1	NR	NR	13.7
Hubbelling et al. 2018 [56]	Retrospective	50	NSCLC	Intracranial	20–37.5/10–15 SRS: 10–22/1	Concurrent Sequential	Nivo; Pembro; Atezo	NR	NR	NR	NR	NR	9
Martin et al. 2018 [57]	Retrospective	115	Various	Intracranial	25–30/5 SRS: 18–20/1	NR	Ipi; Nivo; Pembro	NR	NR	NR	NR	NR	NR
Colaco et al. 2016 [58]	Retrospective	180; Lung: 71	Various	Intracranial	15–24/1	Sequential	Anti CTLA-4; Anti-PD-1	NR	NR	9.3	NR	NR	NR
Chen et al. 2017 [59]	Retrospective	260; Lung 157	Various	Intracranial	15–25/ 1–5	Concurrent (*n* = 28) Sequential (*n* = 51)	Anti-CTLA-4 Anti-PD-1	NR	Concurrent: 1y-LC: 88% Sequential: 1y-LC: 79%	Concurrent 24.7 Sequential14.5	2.3	NR	16
Desideri et al. 2018 [60]	Retrospective	20 Lung: 17	Various	Intracranial	20–30/ 5–10 SRS: 18–40/ 1–5	Concurrent	Nivo	NR	NR	12.5	7.0	50%	5.8
Verma et al. 2018 [61]	Retrospective	60 Lung: 41	Various	Extracranial	45/2x/day 54/15 SABR: 50-60/4	Concurrent	Ipi, Pembro	Ipi: 3 mg/kg q3w Pembro: 100 mg q3w	NR	NR	NR	NR	25
18 Studies		1736 patients	Overall Weighted Mean						70.7%	12.4 months	4.6 months	41.3%	20.0%

NSCLC = Non-Small-Cell Lung Cancer; RT = radiation therapy; SABR = stereotactic ablative radiotherapy; SRS = stereotactic radiosurgery; LC = local control; OS = overall survival; PFS = progression free survival; IT = immunotherapy; Pembro = pembrolizumab; Nivo = nivolumab; Atezo = atezolizumab; Ipi = ipilimumab; CTLA-4 = cytotoxic T-lymphocyte-associated antigen 4; PD-1 = programmed cell death protein-1; PD-L1 = programmed death-ligand.

**Table 2:** Summary of Toxicity ≥Grade 3 with RT-ICI combinations in NSCLC.

Study Design	Follow-Up in Months, Median, Range	Toxicity ≥ Grade 3 %, Weighted Median	Most Common irAEs
AntiPD1/antiPD-L1 + SABR	14.0 (2,9–33)	14,5%	Pneumonitis, asthenia, breakthrough pain, colitis, neurological and hepatic toxicity
Anti CTLA4 + SABR	20.0 (2–38)	26,0%	Pneumonitis, fatigue, liver enzymes increase, colitis and neurological
AntiPD1/AntiPD-L1 alone	11.0 (7–14,5)	10,8%	Pneumonia, increased aspartate aminotransferase, skin reactions, pneumonitis, neutropenia, anemia, thrombocytopenia, diarrhea

IT = immunotherapy; RT = radiotherapy; irAEs = immune-related adverse events.
